# A pediatric virtual care evaluation framework and its evolution using consensus methods

**DOI:** 10.1186/s12887-023-04229-1

**Published:** 2023-08-17

**Authors:** Catherine Dulude, Stephanie Sutherland, Shelley Vanderhout, W. James King, Caroline Zuijdwijk, Nathalie Major, Tobey Audcent, Heather Howley, Paula Cloutier, Melanie Buba, Raagini Jain, Justyna Litwinska, Leanne Findlay, Claudia Malic, Karen Macaulay, Kasey Parker, Christine Kouri, Ellen B. Goldbloom

**Affiliations:** 1 Children’s Hospital of Eastern Ontario, 401 Smyth Rd, Ottawa, ON K1H 8L1 Canada; 2 Children’s Hospital of Eastern Ontario Research Institute, 401 Smyth Rd, Ottawa, ON Canada; 3 School of Epidemiology and Public Health, University of Ottawa, 600 Peter Morand Crescent, Room 101, Ottawa, ON Canada; 4 Faculty of Medicine, University of Ottawa, 451 Smyth Road, Ottawa, ON Canada; 5 Children’s Hospital of Eastern Ontario Research Institute Family Leader Program, 401 Smyth Rd, Ottawa, ON Canada

**Keywords:** Virtual care, Evaluation, Pediatrics, Framework

## Abstract

**Background:**

The use of virtual care has increased dramatically in response to the COVID-19 pandemic, yet evidence is lacking regarding the impact of virtual care on patient outcomes, particularly in pediatrics. A standardized evaluation approach is required to support the integration of virtual care into pediatric health care delivery programs. The objective of this work was to develop a comprehensive and structured framework for pediatric virtual care evaluation. This framework is intended to engage and guide care providers, health centres, and stakeholders towards the development of a standardized approach to the evaluation of pediatric virtual care.

**Methods:**

We brought together a diverse multidisciplinary team, including pediatric clinicians, researchers, digital health leads and analysts, program leaders, a human factors engineer, a family advisor and our manager of health equity and diversity. The team reviewed the literature, including published evaluation frameworks, and used a consensus-based method to develop a virtual care evaluation framework applicable to a broad spectrum of pediatric virtual care programs. We used an iterative process to develop framework components, including domains and sub-domains, examples of evaluation questions, measures, and data sources. Team members met repeatedly over seven months to generate and provide feedback on all components of the framework, making revision as needed until consensus was reached. The framework was then applied to an existing virtual care program.

**Results:**

The resulting framework includes four domains (health outcomes, health delivery, individual experience, and program implementation) and 19 sub-domains designed to support the development and evaluation of pediatric virtual care programs. We also developed guidance on how to use the framework and illustrate its utility by applying it to an existing pediatric virtual care program.

**Conclusions:**

This virtual care evaluation framework expands on previously developed frameworks by providing additional detail and a structure that supports practical application. It can be used to evaluate a wide range of pediatric virtual care programs in a standardized manner. Use of this comprehensive yet easy to use evaluation framework will inform appropriate implementation and integration of virtual care into routine practice and support its sustainability and continuous improvement.

**Supplementary Information:**

The online version contains supplementary material available at 10.1186/s12887-023-04229-1.

## Contributions to literature

- The proposed methodology structured within this framework supports and expands on previously published virtual care evaluation frameworks by providing additional detail including evaluation sub-domains and a structure that supports practical application.
- The framework presented herein is widely applicable and can be used to evaluate a range of pediatric virtual care programs in a standardized manner.
- Application of this comprehensive evaluation framework will inform appropriate implementation and integration of virtual care into routine practice and support its sustainability and continuous improvement.

## Background

The global SARS CoV-2 (COVID-19) pandemic accelerated the growth and “normalization” of virtual health care, [1–4] which encompasses any interaction between patients and/or members of their circle of care, using a range of technologies and applications, that support synchronous and asynchronous care delivered at a distance. Examples of virtual care include telehealth, telemedicine, eMental Health and eHealth [4]. Virtual care has been shown to offer benefits to patients, families, and healthcare providers, such as convenience, improved care accessibility, and higher patient and family satisfaction [5]. However, there is limited evidence, particularly for pediatrics, of the impact of virtual care on patient health outcomes and care experiences.

Patients, families and healthcare providers have expressed enthusiasm for virtual care and expect it to continue beyond the COVID-19 pandemic [6]. To ensure appropriate integration into safe, effective, equitable, and cost-effective care, there is a need for evaluation, performance measurement and monitoring of virtual care delivery models [7]. Virtual care is not interchangeable with in-person care. In some cases, the need for in-person care is indisputable; for example, a new patient requiring a physical exam or procedure. In other cases, it is clear that virtual or hybrid in-person/virtual care has benefits; for example, when sub-specialists provide care virtually to remote communities to support assessments or treatments that are not locally available. Yet in many cases, competing factors such as patient or provider preference, access to appropriate technology, and prohibitive travel costs, make direct comparisons between virtual and in-person care difficult. In these cases, establishing whether virtual care is more, or less, appropriate requires evaluation of many factors. A lack of standardized data definitions and evaluation approaches makes evidence synthesis challenging [7, 8]. In the absence of data-driven recommendations and protocols, expert groups have convened to provide guidance on how to safely incorporate virtual care into their healthcare delivery models [9–13].

Evaluation frameworks can be used to guide the evaluation process [14–16] and consist of the following elements: evaluation questions and associated measures or indicators of success, data sources, data collection strategies, and bases of comparison (internal and/or external, standards, etc.); see Additional file 1. A structured framework can be used to support ongoing assessment of health care delivery, determine the value of virtual care for key stakeholders, and facilitate appropriate integration of virtual care into service delivery [10, 17]. While several different evaluation frameworks [10, 18, 19] and measurement standards have been introduced to guide assessment of virtual care, a standardized approach is needed to yield meaningful evidence to guide implementation and integration of virtual care in pediatric settings. The American Academy of Pediatrics (AAP) Section on Telehealth Care’s SPROUT (Supporting Pediatric Research on Outcomes and Utilization of Telehealth) recognized this need and published the SPROUT Telehealth Evaluation and Measurement (STEM) framework [10] with an intention to welcome collaboration towards its continued evolution [20]. The STEM framework synthesizes quality-focused evaluation frameworks and guidelines developed by the National Quality Forum (NQF), [21]. World Health Organization (WHO) [19] and Agency for Healthcare Research and Quality (AHRQ) [22] into a single outcomes-focused framework organized into four measurement domains: health outcomes, health delivery (quality and cost), experience, and program implementation and key performance indicators (KPIs). It is a helpful guideline and clearly aligned with the quadruple aim (improving patient experience, health of populations and provider satisfaction while reducing costs) [23, 24].

We saw an opportunity to build upon the STEM framework and provide additional detail that would support clinical or program leaders in planning and conducting their own evaluations of virtual care. Using the STEM framework [10] as a guide, our aim was to develop a comprehensive framework to support a standardized approach to virtual care evaluation across a variety of programs. We further aimed to create a virtual care evaluation framework that was flexible for use, in both research or quality improvement contexts, at various stages of program development, from planning through implementation, monitoring and improvement.

## Methods

### Setting and team

At the onset of the COVID-19 pandemic, our pediatric academic centre quickly adapted to virtual care delivery in almost all clinical areas with a focus on safety, access, and sustainability. For example, we now deliver approximately 40% of annual outpatient visits virtually, with program-level virtual care evaluation projects, and separate centre-wide initiatives to collect feedback from patients, families, and healthcare providers [25]. However, a comprehensive evaluation approach has not been standardized across the organization.

Thus, we formed a multidisciplinary team of virtual care leaders and stakeholders to develop a unified approach to virtual care evaluation. This initiative was sponsored by the centre’s executive leadership and research institute. The team consisted of 17 clinicians, researchers, operational leaders and stakeholders from diverse clinical and operational specialties: pediatric mental health (PC); pediatric complex care (NM); pediatric surgery (CM); pediatric emergency medicine (RJ); pediatric medicine (MB, WJK, TA); pediatric endocrinology (EG, CZ); autism/behaviour services (JL); quality improvement (CM, MB); epidemiology (WJK); information technology & informatics (KM, WJK, EG); human factors (CD); business intelligence and reporting (KP); program evaluation and qualitative methodology (SS); research development (HH); patient engagement (EG, MB, LF); family voice (LF); equity, diversity inclusion & Indigeneity (CK); social determinants of health (CZ, TA) and medical education (CZ, TA, RJ).

### Approach

**Fig. 1:**
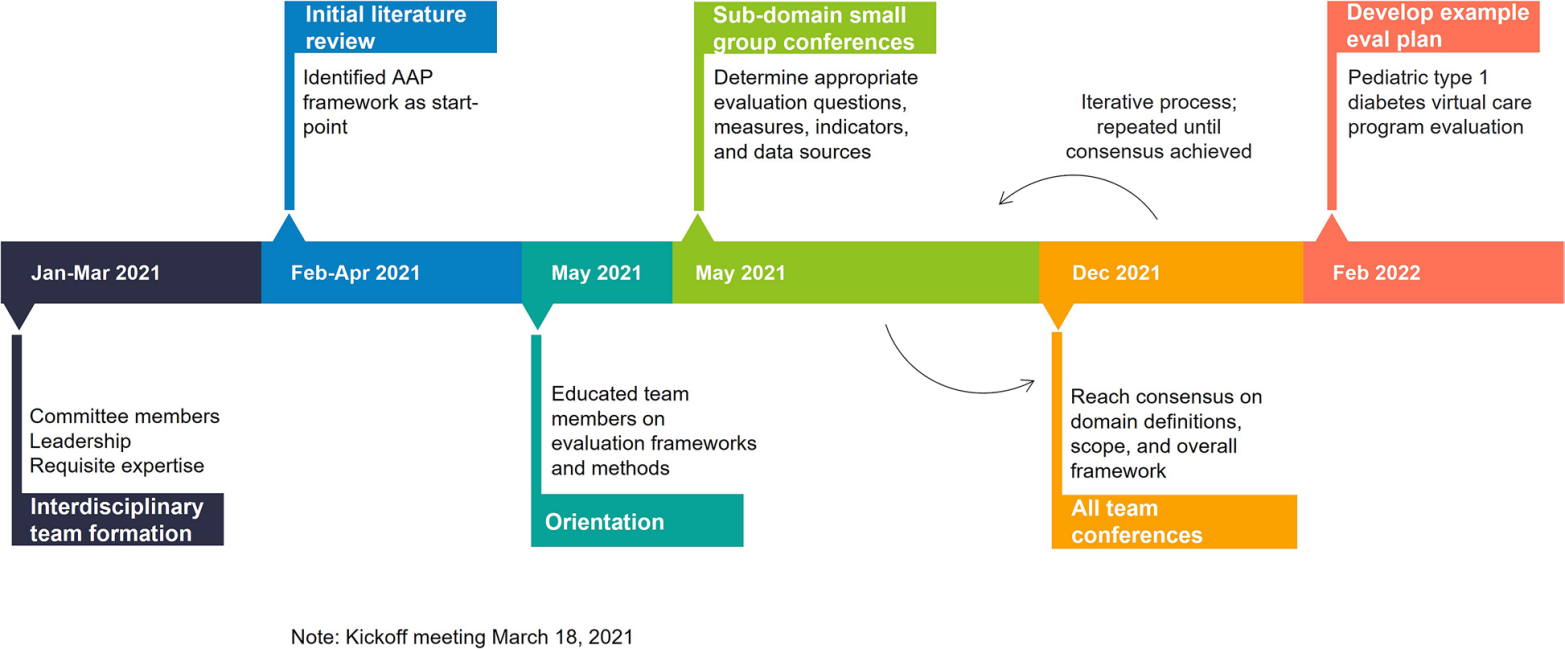
Framework development timeline

We used consensus methodology, including consensus development panels, to produce a pediatric virtual care evaluation framework. The framework development timeline is presented in Fig. 1. A consensus development panel is one of the three approaches (nominal group process, consensus development panels, Delphi technique) to conducting consensus methodology research. We selected consensus development panels as it was most amenable to our objectives by allowing the flexibility to have as many consensus building rounds as necessary. Consensus development panels (also known as consensus development conferences) are organized meetings of between eight and twelve experts from a given field, or combination of fields [26]. However, panel composition can be modified to accommodate larger or smaller groups of experts. In this case teams were comprised of multidisciplinary experts. Consensus development panels are useful for achieving consensus in health care because it is an evidence-based and multidisciplinary approach for problem solving and policy development [26–29].

The STEM framework [10] was critically appraised by all team members and approved as a starting point to populate the domains and sub-domains of our framework. Strengths and weaknesses of the STEM framework for application and use within our context were discussed. To achieve a shared vision of a more extensive, comprehensive, applicable and usable framework, members also used other healthcare evaluation frameworks, including: Ontario Health Quality, [18] COMET Outcomes Classification, [30] Canada Health Infoway Benefits Evaluation Framework (CHIBEF), [31] and other work on the evaluation of telehealth usability [18, 19, 32].

### Consensus on evaluation framework domains and sub-domains

Starting with the four AAP framework domains (health outcomes, health delivery, experience, program implementation and KPIs), multi-disciplinary sub-groups were convened to generate evaluation questions, measures, and data sources for each. Consensus development panels were composed based on interest and expertise, while ensuring representation from diverse clinical areas (e.g., the health outcomes group was composed of a clinical researcher in mental health (PC), research development manager (HH), complex care physician and clinical lead (NM), and a surgeon and quality improvement lead (CM)). Consensus development panels met regularly over a period of eight months. An iterative process, alternating between meeting in smaller panels to develop framework details and as a larger group to discuss and develop consensus on domain definitions and scope, was guided by an expert in evaluation methodology (SS) and a human factors specialist with experience in virtual care development and evaluation (CD).

Each panel met repeatedly, typically three to five one-hour meetings, until they reached consensus on the scope and examples within their domain. Then, the entire evaluation framework was reviewed and revised by all team members until consensus was reached on final content.

### Target audience and application

The team discussed and reached consensus on appropriate settings and contexts for which the framework could be used and clarified the intended audience. As a final step, we applied the framework to an existing virtual care program (pediatric type 1 diabetes) to assess real-world applicability, consistency, and illustrate practical use. Two pediatric endocrinologists used the framework to specify and contextualize the questions, measures/indicators, and aligned data sources and comparators within each domain and sub-domain to create an evaluation plan relevant for the patient population. A formal evaluation of the implementation of the framework is not presented in this manuscript.

## Results

**Fig. 2:**
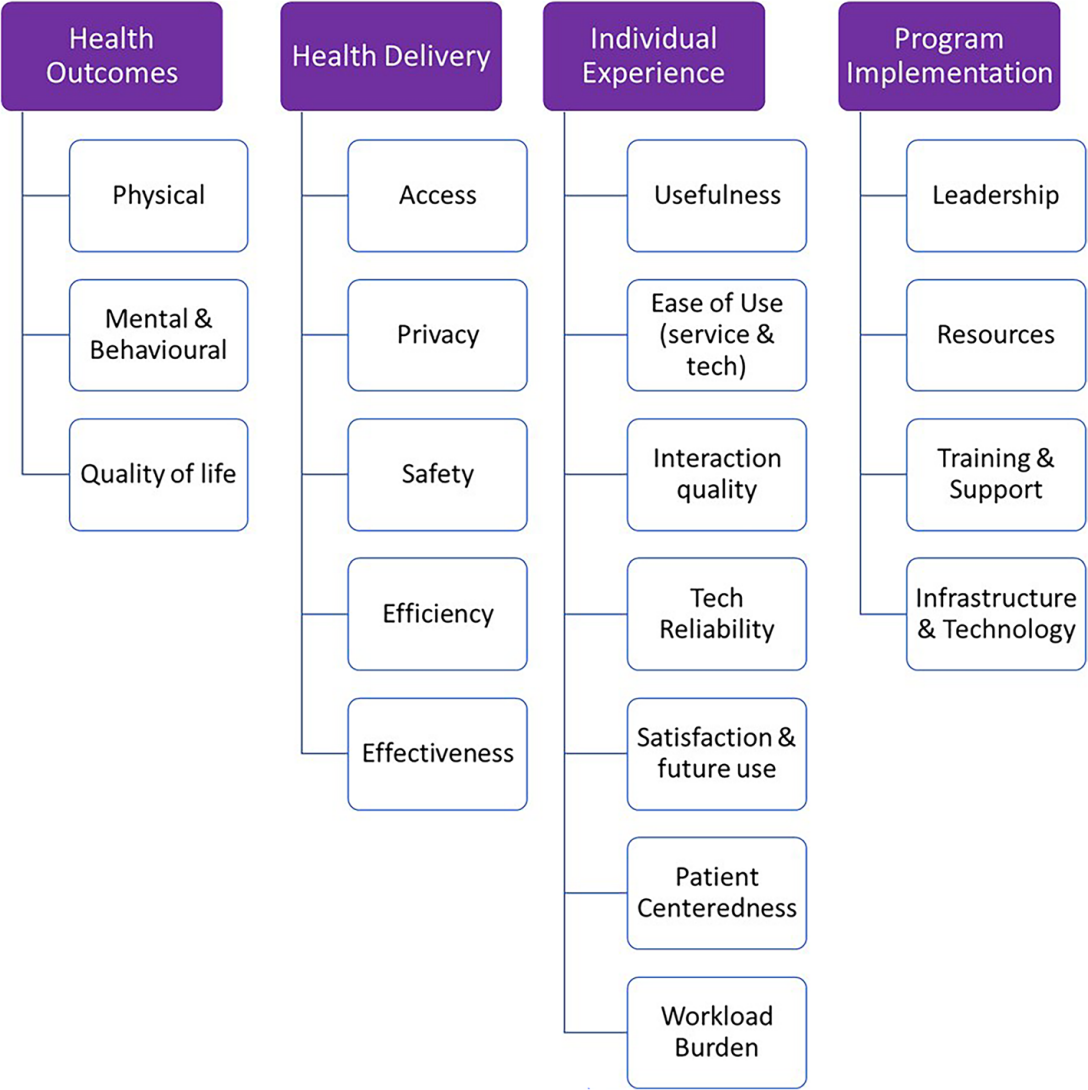
Pediatric Virtual Care Evaluation Framework

We developed a pediatric virtual care evaluation framework (Fig. 2) based on the four domains proposed by STEM [10] and other established evaluation frameworks and tools [17, 29, 31]. Team members agreed the framework should support comprehensive evaluation of virtual care programs and appropriate integration of virtual care based on patient factors (e.g., age, diagnosis, socioeconomic status), timing in the care pathway, and mode of delivery. We also agreed to use the term "virtual care" [3] rather than telehealth or telemedicine to highlight the applicability of this framework to all types of virtual care regardless of technology or synchronicity. We believe this framework can be applied in a variety of contexts ranging from community-based practice to multi-site health centres, and oversight or funding agencies (e.g., government health ministry).

### Framework structure

Our framework has four measurement domains that parallel STEM: health outcomes, health delivery, individual experience, and program implementation. Under these four domains, we have specified 19 sub-domains. In addition to the sub-domains referenced in the STEM domain descriptions and examples, we added the following: Behavioural to the Mental sub-domain under Health Outcomes, Privacy under Health Delivery, Usefulness under Individual Experience, and Leadership under Program Implementation. Under Program Implementation, we also sub-divided System Changes into Resources, Training and Support, and Infrastructure and Technology. With these modifications, we provide a structure that can readily support development of organization-wide or program-specific evaluation questions to illustrate scope, and associated measures, data sources, and bases of comparison for each (see Tables 1, 2, 3 and 4 for examples and Additional file 2 for the full framework). We therefore removed Key Performance Indicators (KPIs) from the Program Implementation domain title used by STEM, as the development of indicators is embedded in the framework structure across all domains. Using this tool, other teams can apply the framework to a variety of patient care settings; articulate their own evaluation questions under each subdomain, and identify measures, data sources and bases of comparison that can be used to answer each question.

**Table 1:** Example for the Health Outcomes domain, Physical health outcomes sub-domain: questions, measures, and data specifics

Health Outcomes				
Example evaluation questions	Potential measures	Data sources	Collection strategy	Basis of comparison
**Physical health outcomes **(*i.e., measures of physiological function, signs and symptoms, laboratory (and other scientific) measures relating to the function of major organ systems, the extent of disease or disability and the provision and/or response to therapeutic interventions)*				
What is the impact of virtual care on individual physiological/clinical measures of health?	Diabetes patient's Hemoglobin A1C (e.g., value, % within target), BMI (e.g., within normal range), Mortality, SSI (Surgical Site Infection) (e.g., SSI infection occurrence, frequency, rate) Complex care pain scores (child or parent proxy, e.g., Linear VAS), feeding/swallowing performance	Patient charts	Quantitative, surveys	Data registries, literature
What is the impact of virtual care on population level physiological/clinical measures of health?	Children/Youth with diabetes: Hemoglobin A1C (e.g., % of patients with A1C within target), BMI (% of patients with BMI SDS > 3; % of patients with increase in BMI SDS), Surgical patients: SSI (% of patients with SSI), mortality	Patient charts	Chart review, reporting tools, quantitative	Regional or provincial data registries (e.g., Canadian Institute of Health Information), established patient registries, literature

*BMI* Body Mass Index

*CIHI* Canadian Institute of Health Information

*SDS* Standard Deviation Score

*SSI* Surgical Site Infection

*VAS* Visual Analog Scale

**Table 2:** Example for the Health Delivery domain, Privacy sub-domain: evaluation questions, measures, and data specifics

Health Delivery				
Example evaluation questions	Potential measures	Data sources	Collection strategy	Basis of comparison
**Privacy**				
To what extent do patients feel comfortable sharing openly with their provider during virtual visits? (e.g., as a function of the patient or provider's home/work environment)	Patient and provider perception	Patient/provider surveys	Post-visit questionnaire	Literature review, other virtual care programs (e.g., mental health), in-person surveys
To what extent can we adhere to privacy and confidentiality standards?	Reported privacy breaches, privacy impact assessments, provider surveys	Safety reporting system, privacy breaches, privacy office audit/ assessments	Quantitative	National/ organizational privacy standards

**Table 3:** Example for the Individual Experience domain, Ease of use sub-domain: evaluation questions, measures, and data specifics

Individual Experience (patient, caregiver, provider, support staff)				
Example evaluation questions	Potential measures	Data sources	Collection strategy	Basis of comparison
**Ease of use**				
To what extent is the process for booking virtual care services easy to use?	Proportion of participants who felt booking virtual care was easy to use, # of deviations from standard booking process	Post-encounter questionnaires/ patient/provider/ clerk experience surveys, support calls, electronic booking system	Qualitative & quantitative	Satisfaction with in-person booking process
To what extent is the method of connecting (to patient/provider) easy to use?	Participant perceptions (patients, caregivers, providers), # of reported errors, # of support calls	Post-encounter questionnaires/ patient experience surveys	Qualitative & quantitative	Literature, established/ comparable telehealth programs

**Table 4:** Example for the Program Implementation domain, Infrastructure and technology subdomain: evaluation questions, measures, and data specifics

Program Implementation					
	Example evaluation questions	Potential measures	Data sources	Collection strategy	Basis of comparison
**Infrastructure & Technology (functionality, performance, security)**^**a**^					
	To what extent has cybersecurity been reviewed and considered? (e.g., has a threat risk assessment (TRA) been conducted?)	# of access issues, adherence to cybersecurity standards, # of threats or security breaches	Cyber-review by 3rd party, TRA, cybersecurity monitoring tools	Quantitative	Applicable regional or national security guidance or directives (e.g., Canadian Centre for Cybersecurity guidance & directives)
	Do providers and/or patients have appropriate access to information required to support care delivery? (e.g., access to whole patient chart, ability to communicate with other providers, or provide follow up information to patients)	Surveys to providers, staff, patients/families, safety reporting system, # of release of information requests	Surveys	Qualitative & quantitative	Other similar programs
	To what extent does the technology support virtual care service delivery?	% of planned/unplanned downtime, # of requests for change/optimization, volume of technical issues, needs assessment/requirements evaluation	HelpDesk tickets/IT reports, project intake/enhancement requests, post-encounter questionnaires	Quantitative	Other similar programs

*TRA* Threat Risk Assessment

^a^Note privacy is under health delivery

### Framework domains and sub-domains

The first domain, Health Outcomes, includes clinical measures of individual and population health within three sub-domains: physical, mental and behavioural, and quality of life (QoL). This domain aligns with the ultimate goal of helping children and youth achieve their best life by going beyond direct measures of health to include the impact of health status on the quality of life of patients and caregivers. We added “behavioural” to the mental health sub-domain to ensure that our framework is relevant for programs delivering developmental and behavioural health services (e.g., applied behaviour analysis, complex care) and provides a holistic view of the impact of virtual care on pediatric health outcomes. Data sources are primarily patient charts, assuming patient and caregiver self-reports and other measures of QoL are included therein. Comparators include internal or external databases or clinical practice guidelines (depending on the evaluation questions). Table 1 provides example evaluation questions for the physical health outcomes sub-domain along with associated measures, data sources and bases of comparison.

The second domain, Health Delivery, includes five sub-domains: access to care, privacy, safety, efficiency, and effectiveness of care delivery. We have defined the scope of these sub-domains as follows. Access to care considers both timeliness (e.g., time to first visit, wait times) and equity (e.g., to what extent the program provides equitable access to care or addresses access or equity gaps present in other programs). Privacy examines the patient environment and the extent to which the tools and processes used to deliver virtual care adhere to privacy and confidentiality standards. Safety considers the potential for virtual care to introduce or address safety risks (e.g., adverse event reporting or risk assessments). Efficiency considers both time and cost (e.g., the financial and time–cost-difference for patients to travel to the hospital compared to attending a virtual visit). Finally, effectiveness considers how well the virtual care program delivers care as intended in terms of quality and quantity, and can be linked back to program objectives and compared to in-person care as appropriate. While organized slightly differently, four of these five sub-domains are consistent across AAP STEM, [10]. Ontario Health Quality [18] and CHIBE [31] frameworks. We added privacy because it is a theme that appears regularly in telehealth experience surveys, especially when evaluating eMental Health programs [33–35] and can be impacted by virtual models of care delivery. Examples of evaluation questions, measures, and data sources for the privacy sub-domain are presented in Table 2.

The third domain, Individual Experience, includes seven sub-domains: usefulness, ease of use, interaction quality, technology reliability, satisfaction and future use, patient-centeredness, and workload burden. To ensure a holistic evaluation of any virtual care program (implemented or envisioned), patient, family/caregiver, provider, and support staff experiences are all considered essential components of high-quality care [7, 24]. To support consistent and comprehensive evaluation of the individual experience, the defining components of usability and themes that appear in validated survey tools (e.g., Telehealth Usability Questionnaire [36]) and CHIBE [31] are represented within the sub-domains. Usefulness is the degree to which a system or tool supports a desired function or goal (e.g., perception of how well the virtual care program supports access to care, clinical outcomes or reduces cost), [37] while ease of use speaks to how easy it is to use, regardless of utility. Interaction quality is the quality of the interpersonal interaction between patient/caregiver and provider that is facilitated by the virtual care system/tool, for example how well participants felt they were able to see, hear, and express themselves [36]. In alignment with the quadruple aim, [23, 24] sub-domains also include patient centeredness (e.g., whether patients should receive care in-person or virtually based on need or personal choice) and individual workload burden (e.g., patient and/or caregiver, provider workload or wellness). Table 3 presents example evaluation questions for the ease-of-use sub-domain along with associated measures, data sources and bases of comparison.

The fourth domain, Program Implementation, highlights key factors impacting system change and sustainability, and includes four sub-domains: leadership engagement and structure (institutional buy-in, provision of policy or guidance), resources (human and financial), training and support (availability and appropriateness), and infrastructure and technology (functionality, performance, and security). The last two sub-domains reflect the CHIBE framework [31]. Here, infrastructure and technology considerations relate to information systems and device capabilities rather than a technology usability evaluation, which falls under the individual experience domain. These four sub-domains are considered important to facilitate planning for implementation and to support decision-making regarding additions or changes to the reach and depth of virtual care programs over time. This domain does not emphasize the identification of KPIs and value definitions as in STEM, [10] because our framework is designed to identify measures and bases of comparison for each sub-domain. Once measures have been identified, these can be used as a repository from which to identify program-specific KPIs. Example evaluation questions, measures, data sources and bases of comparison for the infrastructure and technology sub-domain are presented in Table 4.

### How to use this framework

The first step in using this evaluation framework is to define the objectives of the virtual care program. Excellent resources are available outlining how to develop program objectives. For example, program leaders could consider developing SMART aims following quality improvement methodology [38, 39]. The next step is to develop evaluation questions for each sub-domain that speak directly to the objectives of the virtual care program. For example, when selecting health outcomes evaluation questions, consider whether health outcome targets and impacts should be defined at the individual or population level, or both. For each evaluation question, identify measures that can be used to answer the question (e.g., hemoglobin A1C value as an outcome measure for children and youth with diabetes). Measures may come from encounter records (e.g., observations documented by healthcare providers) or patient-reported outcomes. The operational definitions of each measure should be described so data retrieved by different operators, processes and organizations are consistent and reproducible. Identify existing data sources to determine what data are already available versus need to be collected (e.g., electronic health record data or patient/provider survey results). For each source, consider an appropriate data collection strategy (e.g., qualitative, quantitative, or mixed methods) and the bases of comparison that can be used to determine success. Bases of comparison include an in-person care program, a comparable virtual care program (internal or external), clinical norms, or clinical guidelines as appropriate.

One of the key attributes of evaluation quality is feasibility. This framework provides an organized approach to deciding on outcomes of interest for different program stages (see WHO guidance [19]), priorities, and durations of evaluation (e.g., a program duration may be too short to assess health outcomes, but sufficient to assess program implementation). To demonstrate framework feasibility and how evaluation questions and measures tie back to specific program objectives, two pediatric endocrinologists applied the framework to an existing pediatric type 1 diabetes virtual care program (see Additional file 3). This allowed us to explore how the evaluation framework supported development of a program-specific evaluation plan. They appreciated the detailed guidance the framework provided, felt it was helpful in guiding a comprehensive and systematic evaluation plan, and that it was easy to use. The breadth of the 19 subdomains was deemed to cover all potential areas of interest for evaluation. This exercise also highlighted the need to consider which sub-domains are most relevant in unique contexts. While the pediatric type 1 diabetes virtual care program example illustrates a comprehensive evaluation plan for one clinical area, and the framework’s utility in developing program-specific evaluation questions and measures that tie back to program objectives, we recognize not all programs will have access to robust data sets for benchmarking or measurement. Furthermore, it is not practical for every evaluation to address all sub-domains of the framework in a single initiative. Thus, we believe it is important to consider all domains when scoping out an evaluation, but also that discrete elements of this framework can be used as required, depending on evaluation objectives, audience, and resources.

## Discussion

Herein, we present a comprehensive virtual care evaluation framework that can be used by clinicians, researchers, and program leaders to guide evaluation of a broad range of virtual care programs and services at various stages of development, from planning through implementation and optimization. We describe its development by a multidisciplinary team using consensus methodology and provide instructions for use. Using consensus development panels was an efficient way to obtain results, as developing an evaluation framework was new to many team members due to their clinical and operational (rather than methodological) expertise. Moving forward, team members will be able to apply this knowledge to support development of program-specific virtual care evaluation plans within our institution.

We believe this framework can be used across a variety of virtual care programs, allowing for evidence synthesis and clear comparison of meaningful results. Using a standardized evaluation framework allows for the development of a common set of indicators and ensures program-specific evaluations can be generalized within and across health centres and clinical programs [40]. Ongoing consideration of all domains and sub-domains, even if the focus of a specific evaluation is only on a few, can support program development, implementation and/or optimization and set the stage to streamline a future evaluation (i.e., if a program is implemented with predetermined meaningful measures and data sources, planned or post-hoc analysis of process and outcome measures is simplified and relevant). We encourage using this framework at all stages of program development to guide continuous quality improvement and monitoring of patient and care delivery outcomes ensuring intended benefits are realized and unintended harm avoided. If the primary objective is a holistic evaluation of a virtual care program, we agree with the recommendation of SPROUT to include at least one measure from each domain [20, 41]. However, we feel our framework can also be useful from a research lens where the focus or objective may be within one domain at a time.

Our proposed framework has several strengths. First, it was developed by a multidisciplinary team with patient and family representation, and varied expertise and perspectives in clinical care, health systems, and evaluation. The consensus method used to develop the framework allowed for ongoing discussion about example questions, measures, and data sources in each domain, which led members to take ownership over the material and improved applicability of the framework to multiple contexts. We built upon existing literature to develop a user-friendly, comprehensive framework that considers nuances of pediatric virtual health care delivery. Second, the structure of this framework integrates the opportunity for benchmarking throughout, by requiring users to identify bases of comparison for each selected measure. Finally, application of this framework to an existing virtual care program demonstrated its utility and adaptability. Given the built-in flexibility of the framework, where sub-domains may be tailored to fit individual contexts, we believe it can be used for evidence synthesis across a variety of virtual care programs and support development of evaluation plans appropriate for either research or quality improvement initiatives. This agility is important given the often-overlapping goals of research and quality improvement projects particularly when evaluating health systems impact. Though this framework was developed for the pediatric context by a team with expertise in pediatric care, users have the flexibility to apply it to non-pediatric contexts by modifying or substituting measures as appropriate.

The main limitation to the work presented here is limited testing. Implementation of the framework has not been formally evaluated. Additional testing within an organization that did not develop the framework, or a program that was not represented in our multidisciplinary team, is required to generate validity evidence and further demonstrate its usability, applicability, and versatility.

This framework does not provide the guidance necessary to conduct an economic analysis that would consider the societal value of a particular virtual care program in the broader context of healthcare delivery. However, tools are available to support measuring the value of pediatric telehealth, [8] which we believe are complimentary.

We recognize that some elements of this framework may be difficult to measure robustly at a program or organization level, and the importance of establishing a standardized framework and measures that can be compared across programs, organizations, and institutions. Specifically, risks associated with virtual care, such as wrong, missed, or delayed diagnoses due to the inability to physically examine patients or inability to address emergencies that arise during virtual encounters, may not be easily measured. In an effort to contribute to this important safety evaluation, two members of our team (EG, SV) are leading a national surveillance project for adverse events related to virtual care [42].

We recognize the dynamic nature of virtual care, where innovative technologies, patient and family preferences and needs, and evidence can change and emerge rapidly. Naturally, our framework will need to be adapted for different settings and updated with advancements in this field. Furthermore, while standardized data definitions will facilitate meaningful comparisons and benchmarking, defining these was beyond the scope of our working group and would require broader stakeholder input.

## Conclusion

Our aim in developing a pediatric virtual care evaluation framework is to support development of a standardized approach for evaluating virtual care delivery models to ensure high quality programs and services. Our proposed methodology supports and expands on previously published virtual care evaluation frameworks by providing additional detail, including evaluation sub-domains, and a structure that facilitates practical application.

We believe this framework will be useful in a variety of healthcare contexts. Future research studying the implementation of this framework is required to generate evidence supporting its utility at the program, organization, and system-level. We anticipate a close collaboration with SPROUT and others towards a unified approach for pediatric virtual care evaluation, development of standardized data definitions, benchmarking, knowledge sharing, and ultimately the best health outcomes for our patients.

### Supplementary Information

**Additional file 1.** Elements of an Evaluation Framework.

**Additional file 2.** Virtual Care Evaluation Framework.

**Additional file 3.** Example Virtual Care Evaluation Plan for Type 1 Diabetes clinic, integrated EHR context.

## Data Availability

Not applicable.

## Abbreviations

AAP: American Academy of Pediatrics
AHRQ: Agency for Healthcare Research and Quality
CHIBEF: Canada Health Infoway Benefits Evaluation Framework
COMET: Core Outcome Measures in Effectiveness Trials
COVID-19: COronalVIrus Disease of 2019
KPIs: Key Performance Indicators
NQF: National Quality Forum
QoL: Quality of Life
SARS CoV-2: Severe Acute Respiratory Syndrome Coronavirus 2
SMART: Specific, Measurable, Achievable, Relevant, and Time-bound
SPROUT: Supporting Pediatric Research on Outcomes and Utilization of Telehealth
STEM: SPROUT Telehealth Evaluation and Measurement
WHO: World Health Organization
