# Supplementary material for: A pediatric virtual care evaluation framework and its evolution using consensus methods
Source: BMC Pediatr. 2023 Aug 17;23:402. doi: 10.1186/s12887-023-04229-1 (PMC10433580; doi:10.1186/s12887-023-04229-1)
Supplement: Supplementary file 1 — Additional file 1. Elements of an Evaluation Framework. [file 12887_2023_4229_MOESM1_ESM.docx]

# Additional File 1 – Elements of an Evaluation Framework

| **Framework element** | **Description** |
| --- | --- |
| Evaluation domain | An area of interest, field of knowledge or activities that should be considered in the evaluation |
| Evaluation question | Evaluation questions are linked to evaluation objectives, specific program outcomes and measures or categories of outcomes and measures. Depending on the type of program/initiative evaluation questions may focus on (but are not limited to):   - Planning and implementation issues (e.g., how well was that program planned out and how well was that plan put into practice? - Attainment of program objectives (e.g., how well has the program met its stated objectives?) - Impact of the program on participants and/or community (e.g., what difference has the program made to its intended targets of change patients/families or community as a whole?)^13-15^ |
| Measures / indicators of success | Refer to the specific point at which goals or objectives have been achieved.^13^ Act as proxies for objectives, for example, # of emergency room visits, emergency room wait time, etc.  Note: Having clear objectives will make the job of selecting indicators easier.^14^ Multiple indicators will be necessary to track the success of a program/initiative. |
| Data sources | Divided into two categories: existing data sources (i.e., sources of data that are already in existence (e.g., hospital performance measures/databases, charts, logs, reports, etc.) should be investigated prior to engaging in the work of creating new data sources (e.g., surveys, interview guides, etc., and new data sources (when necessary, the creation new data collection instruments to obtain data that is not currently captured (e.g., build more fields in electronic health records, surveys, etc.) |
| Data collection strategy | Data collection can typically take 3 forms (database/document review, quantitative and/or qualitative). Database/document review would entail, for example, using an existing hospital performance measure (i.e., electronic health record) to track clinic wait times. Whereas, a quantitative strategy would entail, for example, creating a survey to obtain provider perceptions of virtual care. |
| Basis of comparison | Utilized when a baseline is not available or feasible and important for analysis. Comparing findings against established reports (literature) or existing hospital published metrics. |
